# Conserved E2F mediated metastasis in mouse models of breast cancer and HER2 positive patients

**DOI:** 10.18632/oncoscience.259

**Published:** 2015-11-10

**Authors:** Jonathan Rennhack, Eran Andrechek

**Affiliations:** ^1^ Department of Physiology, Michigan State University, East Lansing, MI, USA

**Keywords:** breast cancer, E2F, metastasis, HER2

## Abstract

To improve breast cancer patient outcome work must be done to understand and block tumor metastasis. This study leverages bioinformatics techniques and traditional genetic screens to create a novel method of discovering potential contributors of tumor progression with a focus on tumor metastasis. A database of 1172 of expression data from a variety of mouse models of breast cancer was assembled and queried using previously defined oncogenic activity signatures. This analysis revealed high activity of the E2F family of transcription factors in the MMTV-Neu mouse model. A genetic cross of MMTV-Neu mice into an E2F1 null, E2F2 null, or E2F3 heterozygous background revealed significant changes in tumor progression specifically reductions in tumor latency and metastasis with E2F1 or E2F2 loss. These findings were found to be conserved in human HER2 positive patients. Patients with high E2F1 activity were shown to have worse outcomes such as relapse free survival and distant metastasis free survival. This study shows conserved mechanisms of tumor progression in human breast cancer subtypes and analogous mouse models and underlies the importance of increased research into the characterization of and comparisons between mouse and human tumors to identify which mouse models resemble each subtype of human breast cancer.

## Breast cancer as a heterogeneous disease

Breast cancer is an extremely common and deadly disease. With over 200,000 new cases and 40,000 deaths in the United States annually contributed to the cancer, it is the second leading cause of cancer deaths in women. The main cause of these deaths is the ability of the tumor to metastasize to the lungs, liver, bone, and brain [1]. This is reflected in the survival rates of patients diagnosed with or without tumor metastasis. The five year survival rate of a patient without tumor metastasis is over 90% in contrast to a patient with tumor metastasis who only has approximately a 20% five year survival rate[2]. In order to improve patient outcomes, significant research effort must be placed on treating and preventing tumor metastasis.

A defining characteristic of breast cancer is heterogeneity. Tumors from different patients will have a wide variety of tumor growth rates, response to treatment, and metastatic potential. In order to understand the mechanism behind the diversity of characteristics from one tumor to another many multi “-omic” studies such as TCGA and Metabric have begun to profile tumors from a molecular standpoint [3, 4]. Gene expression data has classified tumors into six main subgroups: Luminal A, Luminal B, Basal, Claudin Low, Normal, and HER2 positive [5]. Each subtype has key driving events such as basal breast cancer being largely associated with p53 mutations or Myc amplification, while HER2+ breast cancer is characterized by the amplification/overexpression of the HER2 protein.

The HER2 subtype has been of special interest due to its clinical relevance. Approximately 25% of breast cancer patients have a HER2 amplification event [6, 7]. This causes the upregulation of HER2, a growth factor receptor, on the cell surface leading to uncontrolled cell growth and increased metastatic capability. Despite the aggressive nature of the subtype, there has been success in developing treatment targeted against the HER2 protein. However, these treatments, such as Herceptin [8] and Lapatinib [9], are not effective in all HER2 positive patients. This indicates that there is heterogeneity in the subgroups as well as redundant oncogenic signaling allowing for survival of the cancer cell without the HER2 signaling cascade.

To better understand and predict the activation of key signaling pathways, oncogenic activation signatures were created. These signatures, developed through Bayesian regression analysis and induced expression of a specific oncogenic driver [10–12], have shown key signaling pathways involved in each molecular subtype. As expected, the basal subgroup has low activation of ER and PR while HER2 positive subtypes have HER2 activation. However it is also seen that subsets of each tumor subtype have a specific oncogenic signaling pattern including a subset of Luminal A tumors with high Src activity. The high Src signaling indicates that a subgroup of Luminal A tumors is dependent upon the Src signaling pathway.

## Mouse models of breast cancer

Mouse models have been created to mimic specific oncogenic drivers, such as Src, in hopes to mirror different types of breast cancer to better understand tumor progression that is dependent on a specific signaling pathway. Induction of breast cancer in a mouse model can be accomplished in a number of different manners. These methods include leveraging tissue specific promoters such as MMTV or WAP to drive expression of an oncogene such as Neu [13], or the use of a tissue specific Cre [14] or inducible drug system to create conditional knockouts of tumor suppressors. Other models use a carcinogen induced model such as DMBA treatment. Models have also been created to investigate specific aspects of breast cancer progression including genomic instability through the loss of key checkpoint or repair proteins like p53 [15] or BRCA [16] or tumor metastasis through induction of PyMT [17]. Given the variety of methods to induce tumors as well activation of unique tumor driving pathways, the transcriptional program in each model would be expected to be unique.

## Gene expression profiling of mouse models of breast cancer

**Figure 1:**
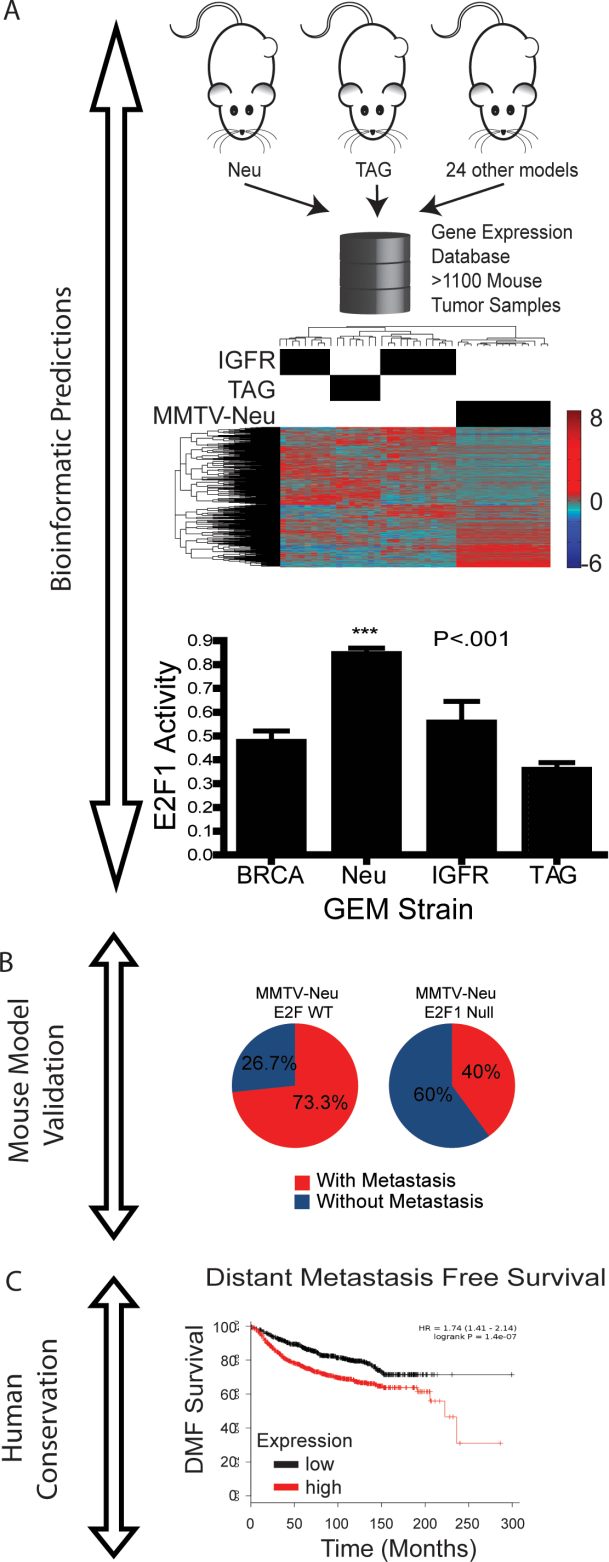
Identification and validation of conserved mechanism of tumor metastasis in mouse models and human breast cancer patients A) A large database of publicly available gene expression data from mouse models of breast cancer was assembled. Clustering revealed key model specific differences. Bayesian regression based signatures were applied to dataset to find activity of key oncogenic signaling pathways. This revealed that E2F1 activity was high in the MMTV-Neu model. B) The functional validation of the finding was completed through traditional genetic screens in which MMTV-Neu tumors in an E2F1 knockout background were found to be significantly less metastatic. C) Extending this to human breast cancer, E2F1 levels were found to correlate with worse distant metastasis free survival times.

To profile this diversity, a database consisting of 1172 tumors from a variety of mouse models was generated [18]. As expected, there was a significant amount of diversity between samples from different models and also within each model (Figure 1A). Despite these differences it was found through unsupervised hierarchical clustering that mouse models of breast cancer clustered into four distinct clusters. These clusters contain transcriptional profiles which regulate different tumor characteristics and are associated with histological patterns such as epithelial to mesenchymal transition (EMT). As expected each of the clusters also had unique oncogenic pathway activation.

Oncogene activation signatures were calculated for each sample in the manner described above, and hierarchical clustering was performed. It was seen that within models sets of tumors had the same signature profile. A key example being the Myc induced tumor models. Tumors derived from these models were extremely heterogeneous [19] and subsets of tumors contained the same oncogenic signaling pattern as tumors from each of the human subclasses of breast cancer [18, 20].

## High E2F activity in MMTV-Neu mouse model

Surprisingly it was noted that the activator subclass of the E2F family of transcription family was seen to be highly active in MMTV-Neu tumor samples (Figure 1A) [21]. The E2F family, classically known to regulate cell cycle [22, 23], has recently been shown to regulate a number of tumor characteristics beyond proliferation such as DNA repair, angiogenesis, and immune-evasion [24–26]. When oncogenic signatures were applied to a group of human breast cancer patients it was seen that a subset of HER2+ patients with unique E2F signaling had worse outcomes, including relapse free survival [21]. This indicates that the E2F family of transcription factors play an important role in HER2 positive tumor progression.

## Loss of E2Fs impact tumor progression MMTV-Neu mouse model

To test the hypothesis that the E2Fs are critical in HER2 tumor progression, MMTV-Neu tumors were crossed into an E2F1 null, E2F2 null, and E2F3 heterozygous background (Figure 1B) [21]. The E2Fs have been shown to be redundant in their binding sites and function, so as expected there was compensation by other E2F family members with the loss of individual E2Fs [27]. Despite the apparent compensation of the E2F knockouts, significant differences were identified in tumor progression between the E2F wildtype and E2F null background indicating specificity in the functions of each E2F family member in regards to tumor progression. There was a significant delay in tumor latency associate with E2F1, E2F2 and E2F3 loss. Furthermore, there was a reduction in tumor burden showing a decrease from an average of 2.5 tumors per mouse in wildtype E2Fs to 1.5 tumors per mouse in the E2F1 null background. The growth rate of the tumors was not affected with E2F2 and E2F3; however, there was a significant increase in the growth rate of E2F1 null tumors. This is likely due to the role of E2F1 in tumor apoptosis.

Striking differences were seen in tumor metastasis. There was a significant reduction in the number of mice with metastasis with the loss of specific E2Fs [21]. In a wildtype MMTV-Neu background it was seen that 73% of mice with tumors develop metastasis to the lungs (Figure 1B). This number is reduced to 40% and 35% with the loss of E2F1 and E2F2 respectively (Figure 1B). It was also seen that the E2Fs affect both early and late stages on metastasis in a cell independent manner. A colony formation assay from circulating tumor cells showed a reduction in the amount of colonies formed in the E2F2 null background indicating a block in the early stages of tumor metastasis. However, the E2F1 null tumors did not show a significant reduction in the amount of colonies formed indicating a block in the late stages of metastasis. The metastasis effects were seen to be background independent with E2F1 null tumors still being non-metastatic when transplanted into a wildtype host.

## Conservation of the E2Fs role in metastasis of human breast cancer

A dataset of gene expression data from human HER2 breast cancer patients was assembled and E2F activity was assessed. It was shown that patients with high E2F1 activity compared to those with relatively lower E2F1 activity had worse metastis free survival [28]. Furthermore patients were separated on the basis of low and high E2F1 activity regaurdless or subtype [29], and it was shown that patients with high E2F1 levels had worse distant metastasis free survival (Figure 1C).

## FUTURE DIRECTIONS

With the establishment of the role of the E2Fs in tumor metastasis, the next goal is to leverage them as a therapeutic target to block metastasis and reduce the mortality associated with breast cancer. It is not predicted that the E2Fs themselves will be good targets for therapy due to their involvement in a myriad of normal cell processes. However, one might predict that there are specific downstream targets of E2F1 or E2F2 that mediate discreet steps in the development of tumor metastasis. As these genes are identified and characterized they may provide opportunities for development as therapeutic targets.

The description of the role of E2Fs in Neu mediated tumors is an example of how an integrative approach can be used to uncover genes that regulate metastasis. As such, this study demonstrates the need for increased basic research into mouse models. In this study we have taken a bioinformatics prediction in a mouse model about the essential nature of the E2Fs in a model, MMTV-Neu. This was investigated and validated through traditional genetic studies, and the role of E2F1 and E2F2 was shown in tumor metastasis. The finding was consistent in HER2 positive patients leading to a potential new therapeutic avenue to block tumor metastasis. To continue studies of this kind, more work must be completed to understand mouse models from a molecular standpoint and to understand which mouse models represent which classes of human tumors. Leveraging advances in bioinformatics and applying them to mouse models of breast cancer therefore presents a unique opportunity to develop and test hypotheses for how metastatic breast cancer progresses.
